# Development of functional foods with stable encapsulated docosahexaenoic acid

**DOI:** 10.3389/fnut.2026.1754055

**Published:** 2026-04-07

**Authors:** Kaumeel Chokshi, Ashfaq Ahmad, Syed Salman Ashraf, Fawzi Banat, Mayssa Hachem

**Affiliations:** 1 Department of Chemical and Petroleum Engineering, College of Engineering and Physical Sciences, Khalifa University of Science and Technology, Abu Dhabi, United Arab Emirates; 2 Food Security and Technology Center, Khalifa University of Science and Technology, Abu Dhabi, United Arab Emirates; 3 Department of Biological Sciences, College of Medicine and Health Sciences, Khalifa University of Science and Technology, Abu Dhabi, United Arab Emirates; 4 Department of Chemistry, College of Engineering and Physical Sciences, Khalifa University of Science and Technology, Abu Dhabi, United Arab Emirates

**Keywords:** bioavailability, DHA stability, docosahexaenoic acid (DHA), encapsulation, functional foods

## Abstract

Docosahexaenoic acid (DHA), an omega-3 long-chain polyunsaturated fatty acid, plays a pivotal role in human health, especially related to neurological development, cardiovascular health, and anti-inflammatory activities. However, its use in functional food products is hindered by susceptibility to oxidation and poor stability during processing and storage. This review describes recent advances in biotechnology to address these issues by using encapsulation methods that can enhance DHA's stability and bioavailability. Firstly, DHA's structural characteristics, food sources, and health advantages are discussed followed by challenges in incorporating DHA into functional foods. Encapsulation offers significant advantages, including enhanced oxidative stability, extended shelf life, controlled release, and improved sensory acceptance, thereby enabling broader application of DHA in diverse functional food products. Different encapsulation strategies, such as microencapsulation, nanoencapsulation, and emulsion systems, are discussed. The review also highlights strategies for enhanced DHA encapsulation as well as applications for functional food focused on microalgae-based DHA products used in foods and raw materials.

## 1 Introduction

In recent years, the food world has witnessed a substantial shift from traditional nutrition to functional foods—foods that yield health benefits above and beyond basic dietary needs. This has been largely due to a heightened consumer awareness of diet's role in disease prevention, aging, and overall long-term health outcomes (1). Among the wide range of bioactive substances being studied for their prospective inclusion in functional foods, omega-3 polyunsaturated fatty acids (PUFAs), and more specifically docosahexaenoic acid (DHA), stand out given their wide array of beneficial physiological effects, which include improvements in cognitive function, cardiovascular health, and anti-inflammatory effects (2, 3).

DHA is a long-chain omega-3 fatty acid naturally found in the brain and retina, making it a vital component of neurodevelopment as well as visual health (4, 5). As endogenous synthesis of DHA in human bodies is found to be inefficient, there arises a need to supplement diet with this fatty acid from sources like fatty fish, fish oil, or algal oil. However, despite the widely documented health benefits of DHA, its incorporation in food products poses substantial challenges, largely due to its chemical instability, poor water solubility, as well as strong fishy flavor and smell—attributes that negatively impact product quality, stability, as well as consumer acceptability (6).

To overcome these issues, encapsulation methods have been identified as an efficient approach to preserve the stability of DHA during processing and storage while masking undesirable sensory properties. Encapsulation is the technique of entrapping DHA in a protective matrix made of diverse materials including proteins, polysaccharides, liposomes, and nanostructures (7). This approach promotes controlled release, improves bioavailability, and enhances oxidative stability. Such technological developments allow the incorporation of DHA into diverse functional food systems.

Current developments in the biotechnology sector have greatly increased the output of stable, plant-based types of DHA, mainly via the fermentation of marine microalgae like *Schizochytrium* sp. and *Crypthecodinium cohnii* (8, 9). Such substitutes have been found to be useful alternatives to fish oil, catering to vegetarian and vegan populations and reducing pressure on aquatic ecosystems. In addition, when combined with microencapsulation and nanotechnology, these bio-derived sources of DHA are increasingly used in the production of next-generation functional foods that ensure both quality and health benefits (10). Several recent reviews have addressed lipid or DHA encapsulation from broader perspectives, including functional lipids, various drug delivery systems, and diverse food bioactives (11–13). These studies do not systematically integrate DHA molecular instability, food-compatible encapsulation technologies, and biotechnological enhancement strategies. The novelty of the present review lies in its DHA-centric and functional food-oriented framework, combining encapsulation technologies with emerging biotechnology approaches and a focused emphasis on microalgal DHA applications. This integrated perspective bridges biotechnology and functional food development and highlights the pivotal role of food biotechnology in promoting human health through enhanced functional nutrition. The scope of this review is limited to food and nutraceutical applications of microalgal DHA, with particular emphasis on its incorporation into functional foods, beverages, and related delivery systems, while pharmaceutical and clinical therapeutic applications are beyond the scope of this review.

## 2 Docosahexaenoic acid (DHA): structure, sources, and health benefits

Docosahexaenoic acid (DHA; C22:6 n-3) is a long-chain omega-3 polyunsaturated fatty acid (PUFA) that plays crucial role in many areas of human physiology, including brain development, vision, and cardiovascular function (14–16). Besides its importance as a nutrient in diet, DHA emerged as a key bioactive lipid molecule, and it continues to gain importance as a functional food ingredient in health-improving food products. Understanding its chemical structure, natural and biotechnological sources, together with its health effects, is important for the efficient use of DHA in functional foods.

### 2.1 Chemical structure and properties

**Figure 1:**
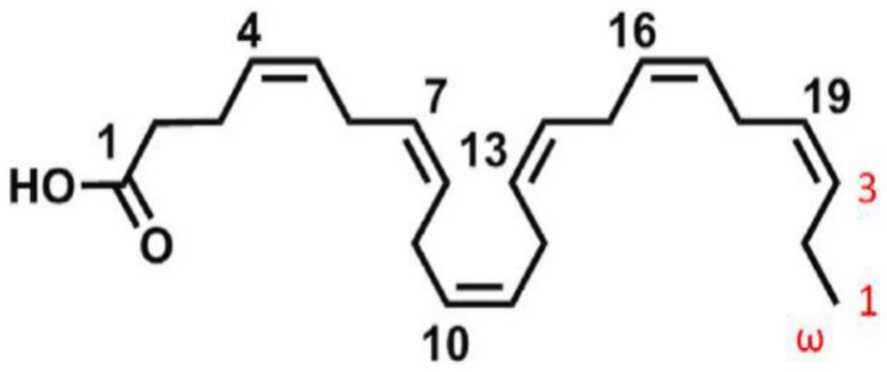
Chemical structure of docosahexaenoic acid [Adopted from (59)].

DHA is characterized by a 22-carbon chain that has six cis double bonds (Figure 1), with the first double bond being at the third carbon from the methyl end, making it an omega-3 fatty acid. The presence of multiple double bonds allows a high level of flexibility and fluidity, which is vital for maintaining the structural integrity of cellular membranes (17), especially in the nervous system and retinal tissues. However, this high level of unsaturation also renders DHA chemically labile and highly prone to oxidation, leading to the formation of off-flavor compounds and nutritional deterioration during processing or storage (18). Such physicochemical properties present challenges to food technologists and highlight the need for stabilization strategies such as encapsulation.

### 2.2 Natural and biotechnological sources

In humans, DHA is primarily obtained through diet, as endogenous synthesis from alpha-linolenic acid (ALA) is not very efficient, with typical conversion rates of less than 1% (19) and varying between approximately 0.2% and 8% depending on factors such as metabolic status and experimental methodology. Traditionally, fish oil derived from fish such as salmon, mackerel, sardines, and tuna has been the most common dietary source of DHA. Fish accumulate DHA through consumption of microalgae, which are the primary producers of omega-3 fatty acids in ocean food chains (20). Due to sustainability concerns and the increasing demand for vegetarian or vegan sources of DHA, the importance of DHA from microalgae has significantly grown. Algal oils, particularly from species like *Schizochytrium sp., Crypthecodinium cohnii*, are rich in DHA and can be produced under controlled fermentation conditions. These sources are free of contaminants such as heavy metals and provide environmentally sustainable sources compared to fish oil. Recent technological developments in biotechnology include the genetic modification of oilseed crops (e.g., *Camelina sativa* or *Brassica napus*) to enable production of DHA (21, 22). Such transgenic crops, which have great potential for large-scale and cost-effective DHA production, have received regulatory approval in specific countries, such as Australia, the United States, and Canada, primarily for use in animal feed, while regulatory evaluation and approval processes are ongoing in other regions.

### 2.3 Health benefits of DHA

DHA contributes to a wide range of physiological functions and have multiple health benefits (Table 1).

**Table 1:** Health benefits of docosahexaenoic acid (DHA).

Health domain	Health benefit	Proposed mechanism of action	References
Integral component of neuronal membranes; promotes synaptogenesis and plasticity	Supports brain growth and cognitive development in infants and children	Integral component of neuronal membranes; promotes synaptogenesis and plasticity	(23)
Cognitive function (Adults)	Enhances memory, attention, and learning; may reduce cognitive decline	Modulates neurotransmission and neuroinflammation	(24, 25)
Cardiovascular health	Lowers triglyceride levels; may reduce blood pressure and heart rate	Anti-inflammatory, anti-thrombotic, lipid-modulating effects	(26, 27)
Ocular health	Supports retinal development and visual acuity	Major structural component of photoreceptor membranes	(103)
Pregnancy and fetal development	Promotes fetal brain and eye development; associated with longer gestation	Maternal-fetal DHA transfer enhances neural and retinal development	(104)
Anti-inflammatory effects	Helps manage inflammatory conditions (e.g., arthritis, IBD)	Precursor of resolvins and protectins that resolve inflammation	(29)
Mental health	May reduce risk or symptoms of depression, anxiety, and ADHD	Modulation of serotonin/dopamine pathways and neuroinflammation	(105)
Immune function	Enhances innate and adaptive immune responses	Modifies eicosanoid production and T-cell signaling	(106, 107)
Aging and neuroprotection	Slows brain aging and reduces risk of neurodegenerative diseases	Reduces oxidative stress and neuroinflammation; supports synaptic integrity	(108)

#### 2.3.1 Neurological and cognitive development

DHA is an important structural element of gray matter in the brain and photoreceptors in the retina, making up 40% of the total PUFAs in specific brain regions and 60% of the PUFAs in the retina. Its importance also includes the neurodevelopment of the fetus and infants (23); moreover, maternal DHA consumption during pregnancy and lactation has been positively related to improved infant cognitive and visual acuity (24, 25). Supplementation in early childhood is linked with better attention, learning abilities, and a lower incidence of neurodevelopmental disorders.

#### 2.3.2 Cardiovascular health

Several clinical and epidemiological studies have shown that DHA provides substantial protection for the cardiovascular system (26) by reducing plasma triglycerides, lowering blood pressure, improving endothelial function, and exhibiting anti-inflammatory effects. It has also been seen to reduce arrhythmia risk, slow atherosclerotic plaque growth, and enhance lipid profiles, especially if taken regularly in an overall balanced diet (27).

#### 2.3.3 Anti-inflammatory and immunomodulatory effects

DHA induces anti-inflammatory properties by acting as a precursor to specialized pro-resolving lipid mediators (SPMs) such as resolvins, protectins, and maresins (28). These molecules regulate resolution of inflammation as well as immune homeostasis. Scientific findings have shown that DHA lowers the prevalence and severity of chronic inflammatory conditions, including arthritis, asthma, and inflammatory bowel disease (29).

#### 2.3.4 Other health benefits

DHA also plays a crucial role in maintaining mental health, as it has been shown to reduce symptoms of anxiety as well as depression, in addition to slowing down age-related cognitive impairment (30) including Alzheimer's. Besides, DHA is also necessary for maintaining eye health, especially its ability to slow down age-related macular degeneration. Moreover, the use of DHA in the field of sport nutrition (31, 32), metabolic control (33), as well as oncology (34) has also emerged recently, though these applications require further research and clinical validation.

### 2.4 Recommended intake and nutritional guidelines

Global health organizations like the World Health Organization (WHO) and European Food Safety Authority (EFSA) recommend a daily DHA intake of 200 to 500 mg for healthy adults, with higher levels advised for pregnant and lactating women to support fetal and neonatal development (35). The recommendations are often given in combination with eicosapentaenoic acid (EPA), another key omega-3 PUFA, though DHA is considered more essential because of its structural and functional roles in the body (36). In summary, DHA is a vital nutritional component with many health benefits and multiple natural and engineered sources. Despite its chemical instability, the growing consumer demand for DHA-rich functional foods highlights the need for greater development of stabilization and delivery systems, particularly through encapsulation methods and biotechnological advances.

## 3 Challenges in incorporating DHA into functional foods

Docosahexaenoic acid (DHA) is a highly valued omega-3 PUFA recognized for its significant health benefits, most notably in neurological, cardiovascular, as well as visual contexts. However, in addition to its substantial therapeutic potential, there are considerable challenges in developing functional foods supplemented with DHA. Such challenges often relate to its physicochemical instability, unpleasant sensory profiles, poor water solubility, as well as incompatibility in different food matrices (6).

### 3.1 Oxidative instability

One of the major challenges in incorporation of DHA into food systems is its high sensitivity to oxidative degradation (37). The presence of six double bonds in its molecular structure makes DHA highly susceptible to oxidation under different factors including oxygen, light, heat, or metal ions. The resulting oxidation process creates lipid peroxides and secondary metabolites in the forms of aldehydes and ketones, which lead to off-flavors, rancid odors, as well as loss of nutritional value (38). Further, these oxidative by-products not only compromise the sensory qualities in the final food product but can also pose a health risk if ingested in high amounts over a prolonged period. The oxidative degradation of DHA poses a challenge in food products with long shelf lives, high processing temperatures (e.g., baking), or poor storage conditions. Exposure to even moderate processing temperatures (45–70 °C), particularly in the presence of oxygen and pro-oxidant compounds, can accelerate DHA oxidation and compromise its retention in long-shelf-life food products (39). Even low levels of oxidation can result in product rejection by consumers, especially in sensitive applications like infant formula or dairy beverages. Thus, resistance to oxidation becomes vital in guaranteeing product quality as well as safety. To prevent oxidative degradation of DHA, antioxidant strategies involving lipid-soluble tocopherols and water-soluble ascorbic acid are frequently applied to interrupt free radical chain reactions, thereby reducing lipid peroxidation and improving the stability of DHA-containing formulations (40).

### 3.2 Low water solubility and dispersion issues

DHA is a hydrophobic compound, which significantly limits its incorporation into water-based food products (41) such as juices, dairy drinks, or soups. Its low solubility in water-based systems can result in poor dispersion, phase separation, as well as sedimentation upon storage. Such physicochemical instabilities can negatively impact the visual appeal, along with texture, of the final product. To address these limitations, emulsification strategies are often used; however, emulsions containing free, unbound DHA remain susceptible to coalescence as well as oxidation upon storage (2). Additionally, the search for stable emulsions that can withstand food processing conditions as well as varying pH levels with preserved bioavailability represents a great technological challenge.

### 3.3 Interaction with food matrices

The incorporation of DHA into complex food matrices offers opportunities for chemical and physical interactions with other components in the system, such as proteins, carbohydrates, and minerals. For instance, even trace levels of metal ions like iron or copper in the matrix can promote lipid oxidation (42), while certain proteins might interact with DHA, thus modifying its release profile or bioavailability (43). In addition, the pH, ionic strength, and water content of the food can have a profound impact on the stability of DHA (44). The matrix effect is particularly pertinent in food items like meat and baked foods, where processing conditions involve high pressures and temperatures. The thermal process can drive the degradation of DHA, especially in the absence of protection, e.g., encapsulation or other stabilization methods. Additionally, protein and lipid transformations during cooking can impact both incorporation of DHA as well as product sensory properties.

### 3.4 Bioavailability and digestive stability

Another significant challenge relates to the bioavailability of DHA after consumption. Even if inclusion in food commodities can increase levels of intake, absorption, as well as metabolic breakdown, can be significantly affected by factors like chemical composition (e.g., triglyceride vs. ethyl ester), presence of other dietary fats, and delivery methods (45). Free DHA is more prone to digestion-related degradation, and without proper formulation a substantial percentage can be lost in the gastrointestinal tract before absorption (46). Encapsulation can be used to increase bioaccessibility by protecting DHA from gastric conditions and increasing its transport to the point of intestinal absorption (44, 47). However, there needs to be careful engineering of encapsulation systems to ensure controlled release, compatibility with enzymatic activity, as well as loss during transport. Additionally, in real food matrices, digestion can also impact the behavior of DHA, requiring *in vitro* and *in vivo* validation.

### 3.5 Regulatory and consumer perception challenges

While many technical challenges related to the use of DHA have been overcome, challenges related to consumer perception and regulatory affairs still remain. The residual fishy smell, even when masked, is likely to deter those who are not familiar with omega-3 products. Vegetarian and vegan consumers might also complain about the fish-derived DHA, thus calling alternative sources like microalgae. Moreover, regulatory bodies in different countries may impose strict limits on oxidation levels, require detailed labeling of source and form, and enforce specific health claim requirements (48). Regulatory requirements also differ substantially across regions. In the European Union, health claims require pre-authorization by the European Food Safety Authority, whereas under DSHEA regulations in the United States, DHA products do not require pre-market approval but must comply with labeling and safety provisions enforced by the U.S. Food and Drug Administration. In several Asian markets, including China and Japan, DHA products are subject to country-specific functional food registration systems that may require product approval and clinical substantiation. In summary, incorporating DHA in functional foods poses a multi-dimensional challenge that involves food science, lipid chemistry, sensory analysis, as well as strict compliance to regulations. Overcoming these challenges requires advancing stability technologies, optimized formulations in terms of the delivery matrix, and improved encapsulation to ensure effectiveness, purity, and acceptability of DHA in real-world applications.

## 4 Encapsulation technologies for DHA stabilization

Encapsulation is a crucial technology used to improve stability, bioavailability, and sensory acceptability of DHA in functional food applications. Because of the high susceptibility of DHA to oxidation, contact with environmental factors such as light, heat, oxygen, and metal ions can cause its rapid deterioration in terms of volatile compound generation, negatively affecting nutritional integrity, safety, and consumer acceptability. Encapsulation technology counteracts these challenges by encapsulating DHA in a protective matrix that provides protection against outside stress factors while promoting controlled delivery (49). Recent advancements have greatly enhanced the encapsulation of DHA, improving its stability and bioavailability in functional foods (Table 2). This section discusses encapsulation material selection, key encapsulation technologies used to stabilize DHA, and few approaches for enhanced DHA encapsulation.

**Table 2:** Overview of approaches for enhanced DHA encapsulation.

Approach	Description	Advantages	Limitations	References
Nano-encapsulation	Nano-sized carriers for DHA protection and absorption	Improved stability, enhanced bioavailability, and targeted delivery	Sensitive to pH and ionic strength	(43)
Spray drying	Emulsified DHA dried into microcapsules	Cost-effective, scalable, and stable microcapsules	Heat-sensitive, oxidation risk	(47, 109)
Pickering emulsion	Particle-stabilized emulsions	Enhanced stability and controlled release	Formulation complexity; limited loading	(70)
Microencapsulation with proteins	Protein-based wall (e.g., whey)	Oxidation protection; functional enhancement	Environmental instability, low oxidation barrier	(58)
Liposomal encapsulation	Phospholipid bilayer systems	Enhanced absorption and protection from degradation	Stability issues, oxidation risk	(110)
Coacervation	Polymer phase separation method	High encapsulation, controlled release	Limited stability in harsh environments	(41)
Electro-spraying	Electric field generates fine DHA particles	Uniform particles, high efficiency	Specialized equipment, formulation sensitivity	(41)
Spray chilling	Cooling-induced solidification of DHA emulsion	Simple, suitable for heat-sensitive compounds	Costly, complex equipment	(58, 111)

### 4.1 Encapsulation material selection

The choice of wall material is critical to the success of DHA encapsulation and should account for several factors such as the food matrix, processing conditions, regulations, and the desired release profile (47). Wall materials must exhibit good barrier properties against oxygen and light, food-grade status, compatibility with processing methods, digestive stability, and release in the intestine, etc. (11, 50). In addition to functional performance, cost considerations play a crucial role in wall material selection, as large-scale industrial application requires economically viable materials that balance encapsulation efficiency, scalability, and raw material availability. Recent research has focused on natural and plant-based encapsulating materials such as pea protein, alginate, cellulose derivatives, and resistant starches to meet clean-label and sustainable product trends. Additionally, co-encapsulation of DHA along with antioxidants like tocopherols or polyphenols can be used to further enhance oxidative stability and create synergistic health benefits (51).

### 4.2 Encapsulation technologies

#### 4.2.1 Microencapsulation

Microencapsulation is a widely used process in the food industry for encapsulation of DHA. The process involves the encapsulation of DHA droplets in wall materials, which upon encapsulation yield microcapsules of size ranging from 1 to 1,000 μm. The most common wall materials used in the process include proteins (e.g., whey protein isolate, casein) (52, 53), polysaccharides (e.g., maltodextrin, gum arabic, inulin) (54, 55), and lipids. Spray drying is a widely used process of microencapsulation, where an emulsion of a wall component and DHA is atomized into a hot drying chamber (56, 57). The rapid evaporation of the solvent allows a powder to be formed, which then leads to the formation of a powder of microcapsules. This method is highly scalable and cost-effective, making it suitable for large-scale industrial production. However, the high inlet temperatures (typically >150 °C) pose a possible risk of partial degradation of DHA unless carefully optimized. Other techniques for microencapsulation include spray chilling (or spray cooling), which uses lipids as wall materials that solidify upon cooling, and coacervation, which relies on phase separation of colloids (typically gelatin and gum arabic) to encapsulate the core material (58). These methods offer better protection for heat-sensitive compounds but may be limited by cost, process complexity, or stability in water.

#### 4.2.2 Nanoencapsulation

Nanoencapsulation refers to the encapsulation of DHA in nanostructures with diameters ranging from 10 to 1,000 nanometers. It provides better protection from oxidative degradation, enhances the available surface area, solubility in aqueous media, and bioavailability, mainly as a result of the improved cellular uptake that is linked with nano-sized particles (47, 59). Techniques such as nanoemulsification, liposome formation, nanostructured lipid carriers (NLCs), and polymeric nanoparticles have been explored for the delivery of DHA. Nanoemulsions are thermodynamically or kinetically stable colloidal systems formed by high shear mixing or ultrasonication. These systems can maintain DHA in a dispersed state while being protected by surfactant-stabilized interfaces. Liposomes, composed of phospholipid bilayers, mimic biological membranes and can encapsulate DHA in their hydrophobic core. They provide biocompatibility, targeted delivery, and better absorption but they are relatively expensive and might require refrigeration (60). Nanostructured lipid carriers (NLCs) combine solid and liquid lipids to form a solid lipid matrix that encapsulates DHA and allows for controlled release (61). These carriers improve load efficiency and oxidative resistance, but they can face issues related to long-term stability. Polymeric nanoparticles, which are based on biodegradable polymers such as polylactic acid (PLA) or chitosan, offer strong encapsulation efficiency along with sustained release properties (62). However, their application in food systems is still in the developing stage due to uncertainties in regulatory frameworks and polymer safety issues. Moreover, their regulatory status varies across regions, with the European Union requiring specific safety assessment and authorization under novel food regulations, the United States evaluating nano-enabled ingredients within existing FDA frameworks, and several Asian countries adopting case-by-case evaluation approaches.

#### 4.2.3 Inclusion complexation

Inclusion complexation involves the non-covalent entrapment of DHA molecules in cavities of host molecules such as cyclodextrins. The cyclic oligosaccharides have a hydrophobic inner cavity and a hydrophilic exterior, making it feasible to trap lipophilic molecules like DHA (63). Complexation with cyclodextrin increases the solubility of DHA in water, removes any unpleasant fishy smell, and provides better protection against oxidative rancidity. Inclusion complexes are formed by kneading, co-precipitation, or freeze-drying (64). The major advantages of such complexes include improved bioavailability and compatibility with dry food matrices (e.g., diet supplements). However, their loading potential can be lower compared to micro- or nanoencapsulation methods, and complex formation can be compromised by fluctuations in pH and temperature conditions.

#### 4.2.4 Emulsion-based encapsulation

Emulsion systems, including simple and multiple emulsions (e.g., oil-in-water and water-in-oil-in-water), have been widely used to deliver DHA in liquid food products (2). These emulsion systems usually include emulsifying agents like lecithin, Tween 80, or whey proteins to promote the stabilization of DHA-containing droplets. Advanced techniques such as Pickering emulsions, stabilized by solid particles instead of surfactants, exhibit better oxidative stability and high potential in terms of clean-label solutions (65). Double emulsions have also been shown to enhance protective functions and enable the co-encapsulation of DHA with other bioactive agents or flavorants (66). The main challenge with emulsion systems is achieving long-term stability to avert phenomena like creaming, coalescence, or phase separation, particularly when exposed to different pH and temperature levels.

### 4.3 Approaches for enhanced DHA encapsulation

Encapsulation technology has emerged as a pivotal innovation in the food sector, enabling the efficient integration and stabilization of bioactive compounds in various food matrices. This advancement supports the formulation of functional foods with superior nutritional profiles, extended storage stability, and enhanced sensory appeal. Moreover, encapsulation facilitates the controlled and targeted release of bioactive, thereby optimizing their bioavailability and physiological effectiveness. It is regarded as a highly effective strategy for enhancing the therapeutic efficacy and stability of bioactive ingredients in the development of health-promoting functional foods (59, 67, 68). DHA is recognized for its antioxidant properties and its ability to reduce hypertriglyceridemia. However, its application in functional foods is restricted due to its poor solubility and vulnerability to oxidation and undesirable sensory properties.

**Figure 2:**
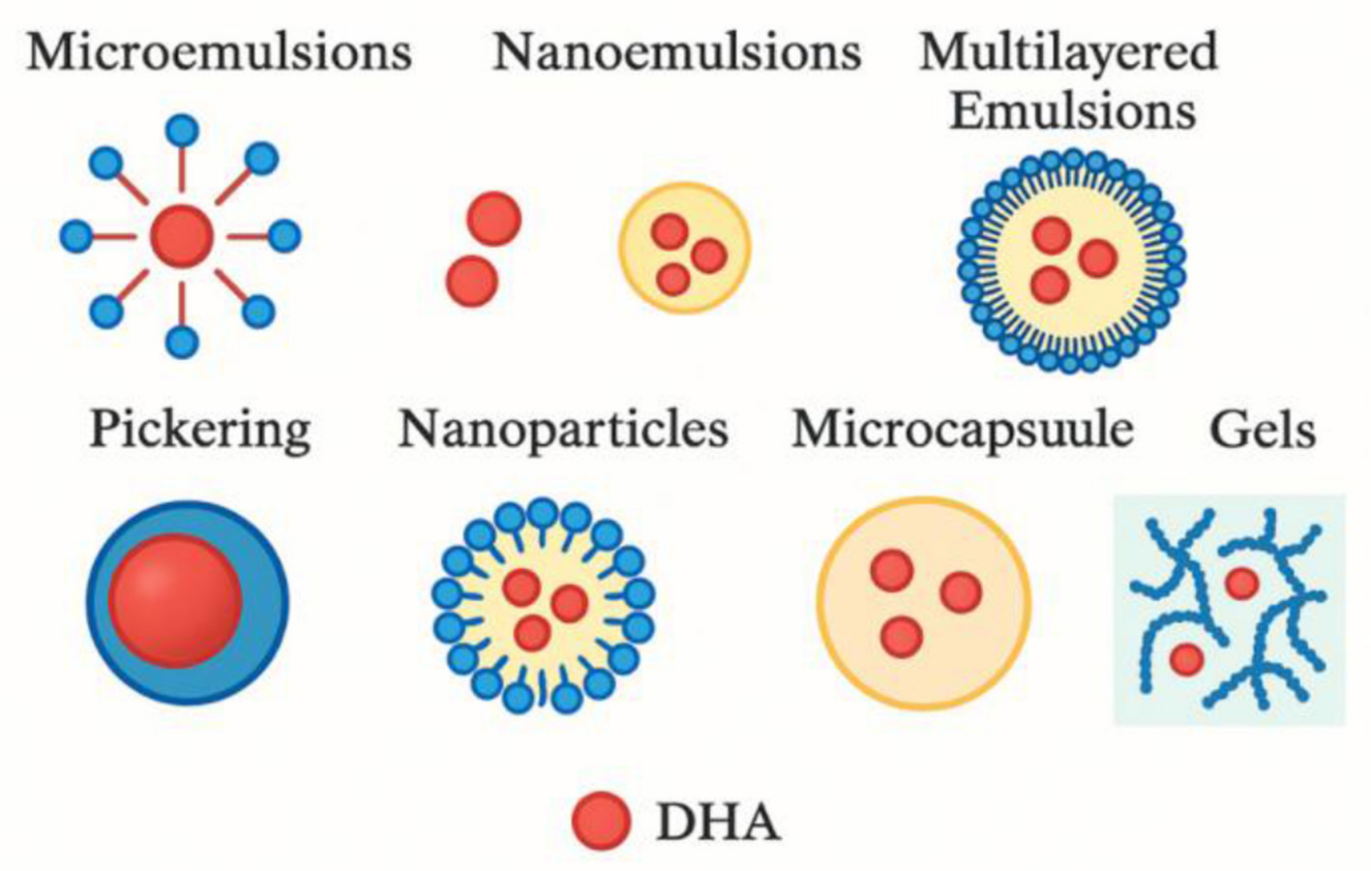
Schematic representation of various delivery systems for DHA (Docosahexaenoic Acid), including microcapsules, microemulsions, multilayered emulsions, nanoemulsions, gels, nanoparticles, and Pickering emulsions. Adopted from Lv and Xu (41).

Figure 2 depicts different delivery systems that indicate possible locations for DHA. It illustrates the structural diversity and encapsulation strategies used to enhance the stability, bioavailability, and targeted release of DHA in functional food and biomedical applications. Among these, nano-encapsulation techniques have garnered particular attention due to their capacity to produce particles with enhanced surface area, superior bioactive protection, and improved gastrointestinal absorption. Researchers have developed nano-encapsulation technique using materials like zein (nano-FO), an edible corn protein that resembles the structure of milk in appearance, to form a core-shell structure (69). The nano-FO formulation has shown promising results, such as an increase in gastrointestinal absorption, an increase in the quantity of nutrients delivered to maternal, fetal, and offspring brains, and a reduction in the accumulation of fatty acids in the fetal liver. Behavioral assessments have indicated that a diet incorporating nano-FO significantly enhances learning and memory compared to a standard fish oil diet (69).

A significant improvement in the stability and delivery of DHA has been achieved with an innovative Pickering emulsion that is stabilized with a glycosylated whey protein isolate-chitooligosaccharide (gWPI-COS)-based encapsulation method for algal oil (70). This emulsion exhibited superior emulsification properties due to strong interactions at the oil–water interface facilitated by glycosylation and COS. The emulsion demonstrated enhanced thermal, storage, and oxidative stability compared to the WPI and WPI-COS systems. It retained its structure after oral and gastric digestion while effectively releasing DHA during digestion in the small intestine. A significant improvement in the bioavailability of DHA in the small intestine was observed, highlighting the potential of this emulsion to effectively deliver DHA into the body (70).

A recent study has investigated the effect of encapsulating DHA within a food matrix on its bioaccessibility, comparing it with its natural unencapsulated form (53). In this study, DHA oil, enriched with DHA-triacylglycerols, was formulated into a Pickering emulsion. Heat-denatured whey protein isolate particles were used in the preparation of this emulsion, which was then incorporated into homogenized liquid eggs to generate omelets using the emulsion. It was found that lipid droplets in omelets containing encapsulated DHA oil were smaller and more evenly distributed compared to those in omelets that did not contain encapsulated DHA oil. During the intestinal phase, encapsulating DHA oil greatly enhanced its hydrolysis by pancreatic lipase, resulting in a more substantial release of free DHA. There was a decrease in DHA esterification within the triacylglycerols when encapsulated DHA oil was used compared to non-encapsulated DHA oil, where 43% of DHA remained esterified within the triacylglycerol. In the case of encapsulated oil, free DHA constituted 52% of the total DHA, whereas in the case of non-encapsulated oil, it was 40%. In addition, the amount of DHA released after an hour of intestinal digestion with encapsulated DHA oil was equal to the amount released after 2 h with non-encapsulated DHA oil, which indicates DHA's significantly enhanced bioavailability (102).

Another study showed that DHA microalgae oil could be encapsulated using Whey protein isolate (WPI) and gum Arabic (GA) through complex coacervation (71). In order to enhance the stability of microcapsules, an EPS-producing strain was introduced to improve the stability of emulsions and oxidation resistance. These microcapsules were studied in terms of their physicochemical properties as well as their stability against oxidation. In the study, EPS++ microcapsules, which contained a 25.83 μm particle size, were found to have good emulsion stability and to be stable in a catalytic process. A scanning electron microscope was used to verify that the surface structure under these microcapsules was uniform, which validated their high encapsulation efficiency of 92.5%. EPS++ provided significant protection against oxidation during storage, exhibiting a low level of peroxygen (PV), headspace propanal, and p-anisidine (p-AV), indicating that the strain is more resistant to oxidative degradation. Moreover, the study concluded that EPS-producing strains are potentially useful wall materials for enhancing the oxidation stability of DHA microalgae oil, making it more suitable for functional foods and applications related to health and wellness (71).

A recent study utilized different proteins, including ovalbumin, myosin, 7S soy globulin, and β-lactoglobulin, which are hydrolyzed by chymotrypsin, to form micelles for the encapsulation of DHA, thereby enhancing its stability and solubility (52). These micelles, confirmed through fluorescence quenching, provided spatial stability and protected DHA from thermal and UV degradation. The uptake of DHA by Caco-2 cells was significantly enhanced, with rates 2.7 to 3.95 times higher than those of free DHA, thereby strengthening cellular antioxidant defenses. Furthermore, in HepG2 cells, these micelles effectively reduced lipid accumulation, indicating their potential as efficient carriers of DHA. This biotechnological approach shows potential for improving the bioavailability and stability of DHA in functional foods, thus providing considerable health advantages (52).

Researchers have developed a plant-based DHA delivery system using emulsion technology, where algae oil droplets were coated with pea protein (PP) and flaxseed gum (FG) (72). Pea protein stabilized the oil droplets, while flaxseed gum was deposited through electrostatic deposition to enhance stability and reduce oxidation. In the PP/FG complex, there were lower interfacial tensions at the oil-water interface than those found in PP alone, suggesting that improved surface activity contributes to emulsion stability by reducing the interfacial tension. Furthermore, an analysis of the volatile compounds demonstrated a significant reduction in the characteristic fishy odor of algal oil due to the absence of heptanal and (E,Z)-3,5-octadiene-2-one levels in the interfacial layer. This odor suppression was linked to the coating's role in minimizing lipid oxidation. As effective carriers for omega-3 fatty acids, these plant-derived emulsions offer the potential of being used in the formulation of functional food and beverages, as they demonstrate promise as superior carriers for omega-3 fatty acids (72).

According to an *in vitro* gastrointestinal digestion model, algae oil containing 42% DHA was studied for the accessibility of bioactive compounds (73). An evaluation of three different delivery systems was conducted in this study: bulk oil, an oil-in-water (O/W) emulsion stabilized with soy protein, and an emulsion gelled with carrageenan. Results showed lipid digestion kinetics were slightly influenced by delivery system type, with lipolysis percentages from 49% to 52%. Lipid oxidation was significantly lower in emulsified oils vs. bulk oil. DHA bioaccessibility was highest in gelled emulsion at 84%, followed by O/W emulsion at 71%, and bulk oil at 58%. The study concluded that emulsified systems improve solubilization and potential intestinal absorption of omega-3 fatty acids (73).

A study investigated DHA nano-capsules made from microalgae oil using carbohydrates, polymers, gum, and proteins as wall materials (47). A stable DHA formulation was produced by spray-drying the DHA solution. The optimized formulation yielded particles with a size of 780 nm, a spherical morphology, 98.5% encapsulation efficiency, and high oxidative stability under storage conditions. A Brunauer-Emmett-Teller (BET) analysis revealed that the surface area of the materials was increased, the pore diameter was larger, and the size distribution was narrower. In *ex vivo* studies, compared to commercially available formulations, the intestinal absorption of DHA was doubled, while *in vivo* tests showed that brain DHA concentrations were 2.9-fold higher than those in pure DHA oil (47). Based on this study, it was determined that the combination of different wall materials improved the bioavailability, shelf life, and oxidation resistance of DHA oil.

The exploration of plant-based proteins and polymers as encapsulating agents has yielded promising outcomes in enhancing the stability and bioavailability of DHA (49). These substances effectively form a protective layer around DHA, which prevents its degradation and facilitates improved absorption in the gastrointestinal tract. In addition, lipid-based delivery systems, such as nanoliposomes and solid lipid nanoparticles, have shown efficacy in encapsulating DHA. These systems shield DHA from oxidative damage and improve its bioavailability by promoting its absorption in the body. Emerging technologies like electro spraying and ultrasonic-assisted encapsulation have also shown significant promise in boosting the stability and bioavailability of bioactives such as DHA. Electro sprayed ultrathin capsules and ultrasonic-assisted protein-polysaccharide complexes offer enhanced protection and controlled release (74, 75). These encapsulation strategies are increasingly applied in functional foods to improve product quality and consumer appeal (12). Thus, biotechnological advancements have opened new possibilities for making functional foods with stable encapsulated DHA, offering considerable health benefits and improving the overall quality of food products.

## 5 Applications of microalgal DHA for functional foods

A wide variety of bioactive compounds have been found to be beneficial to the human body due to the recent advancements in functional food and supplements. Several studies have demonstrated that omega-3 polyunsaturated fatty acids, such as EPA and DHA, have a beneficial effect on a range of neurodegenerative disorders, inflammatory diseases, cardiovascular events, and certain types of cancer. DHA plays a vital role in maintaining the integrity of neuronal membranes and in maintaining the body's health (27, 76).

Microalgae are an exceptionally valuable source of omega-3 fatty acids because of their rapid growth compared to other familiar sources, such as fish, plants, and animals. They can produce up to 10 times more oil than terrestrial oil-producing crops. Moreover, microalgal oil and biomass can be directly utilized as dietary supplements, providing the benefit of lacking a fishy flavor and eliminating the risk of fish-related allergies. Unlike conventional crops, microalgae do not need fertile land for growth, as they can flourish in limited amounts of brackish or seawater (77). One notable advantage of microalgae is their biochemical makeup, which can be readily modified to adapt to different environmental conditions (78). Microalgal biomass is a valuable ingredient that can be incorporated into functional foods to increase their nutritional and functional properties. It contains a high protein content and valuable bioactive compounds like pigments, polyunsaturated fatty acids, amino acids, mineral salts, and vitamins. It can be consumed directly by consumers as a nutraceutical or functional food in powder, capsule, pill, and tablet form. Moreover, microalgae have the potential to significantly improve the nutritional value as well as the texture, such as firmness and fracturability, without affecting the taste or texture of the food (79–82). Microalgal species such as *Schizochytrium* sp., *Porphyridium cruentum, Arthrospira* sp., *Crypthecodinium cohnii, Chlorella* sp., *Haematococcus* sp., *Chlamydomonas* sp., *Euglena gracilis, Dunaliella* sp., and *Ulkenia* sp. have been recognized safe for food applications by the regulatory authorities. These species have been utilized in a broad range of commercially successful biotechnological applications, such as dietary supplements, functional foods, food additives, livestock feed, agricultural inputs, pharmaceuticals, cosmetics, and natural colorants (83, 84).

Table 3 describes microalgae-based DHA products used in foods and raw materials. Due to their rich composition of bioactive compounds, particularly PUFAs such as DHA, microalgae-based products have gained significant attention in the functional food and nutraceutical industries. Various microalgae strains are being utilized across multiple product categories to enhance health benefits while maintaining desirable sensory properties. In the beverage sector, health drinks and infant-grade milk fortified with microalgae strains such as *Chlorella* and *Ulkenia* offer enriched levels of omega-3 fatty acids, providing optimal nutritional support for various age groups (85, 86). In the nutraceutical domain, diverse algal species are processed into powders, capsules, pills, and tablets, providing consumers with convenient dietary supplementation options. Functional food applications often utilize *Arthrospira* and *Chlorella* species as additives or supplements due to their high protein content, bioactive pigments, and PUFAs that contribute to both nutritional and therapeutic benefits (86). Microalgae are also extensively used in animal feed and fodder formulations. Conventional food products can also be fortified with microalgae to improve their nutritional profile without compromising texture or flavor, enabling seamless integration into everyday diets (85). Furthermore, the growing demand for plant-based proteins has also led to the use of microalgae as meat alternatives, particularly vegan patties and protein products.

**Table 3:** Microalgae-based DHA products used in foods and raw materials.

Product type	Microalgae strains	Application	Health benefits	Commercial examples	References
Nutraceuticals	Various algal species	Powders, capsules, pills, tablets	Convenient forms for dietary supplementation	DHA capsules (e.g., DSM-Firmenich life'sDHA^®^, Nordic Naturals Algae DHA)	(86)
Functional foods	*Arthrospira* sp., *Chlorella* sp.	Food additives, dietary supplements	High protein content, bioactive components like pigments, and PUFAs	Nestlé NIDO growing-up formula with DHA, Gerber DHA & Probiotic Single Grain Rice Cereal	(86)
Health beverages	*Chlorella* sp., *Ulkenia* sp.	Fortified drinks, infant-grade milk	Enriched with omega-3 fatty acids for optimal health	Organic Valley^®^ Family First^®^ DHA Omega 3 Whole Milk, Foremost Omega 369 Milk	(85, 86)
Animal feed and fodder	*Schizochytrium* sp.	Substitute for fish oil, fishmeal, and vegetable oils	Increases PUFAs in tissues, improves antioxidant ability, and immune response	Veramaris^®^ algal oil for aquafeed, DSM-Firmenich DHAgold™	(85, 86)
Meat alternatives	*Schizochytrium* sp.	Vegan patties and protein products	Used to enhance the omega-3 profile in plant-based meat	DHA-enriched plant-based meat products; omega-3 fortified vegan foods (emerging market)	(112)

*Schizochytrium* sp., a source of high-quality DHA-rich oils, has received considerable attention for its high DHA content (87). Recent studies have shown that supplementing goats' diet with *Schizochytrium* dry biomass can prevent udder lesions and improve their health, which has also been demonstrated to have positive effects on udder health (88). There was a decrease in both the mean somatic cell count and the number of udder pathogens by 5.34 log cells/mL and 10%, respectively. It has been reported that when milk-fed calves were supplemented, the levels of propionate in the rumen were reduced, dropping from 22.46 to 17.92 mmol/L because of dietary supplementation. It also improves the activity of catalase and glutathione peroxidase, increasing their activity from 4.16 to 11.52 U/mL and from 139.33 to 180.18 mmol/L, respectively. In addition, these changes enhance the calves' ability to produce antioxidants (89). Further, researchers have demonstrated that the addition of DHA-rich *Schizochytrium* dry biomass to livestock and poultry feed increases the level of polyunsaturated fatty acids and enhances the flavor of tissues, meat, milk, and eggs, along with other nutrients. For example, there has been a 1.4% increase in DHA levels in chicken thigh meat, and nearly two-fold increase in lipid species that have five or more double bonds in pig muscles, as well as an 11.4-fold increase in DHA levels in male Qaidamford cattle (90, 91).

In another study, *Diacronema vlkianum*, which contains 101 mg/kg of EPA + DHA and is equivalent to 24.2 grams of microalgae for humans, was found to increase fatty acid concentrations in various tissues and in the bloodstream (92). It has also been demonstrated that supplementation of DHA from *C. cohnii, Schizochytrium*, and fortified snack bars is bioavailable in similar amounts, with a clear dose-response relationship across these forms of supplementation (93). Based on the comparable plasma levels of DHA between capsules and fortified snack bars, it may be possible that consuming DHA in food may increase its absorption in the body.

**Figure 3:**
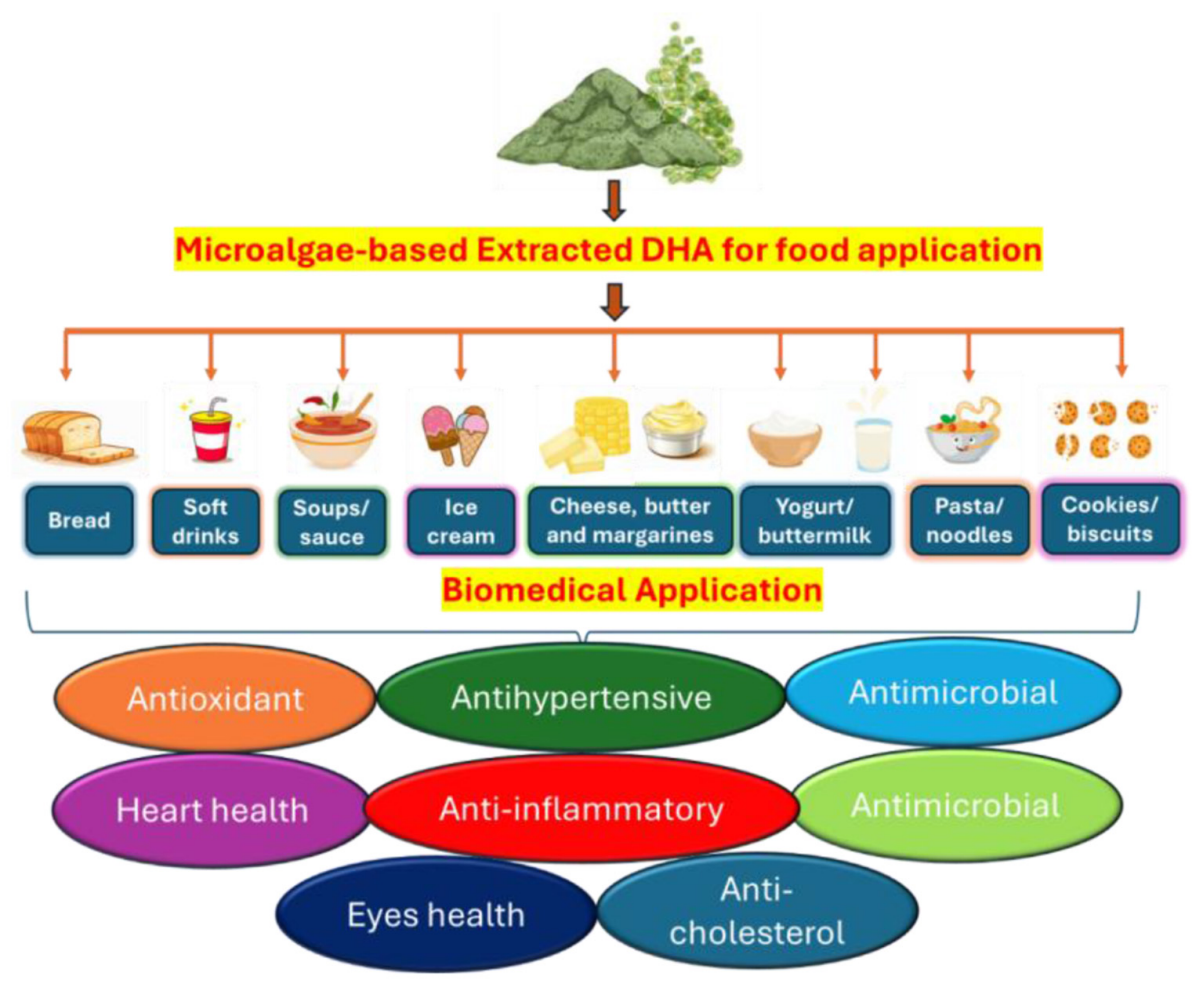
Applications of microalgae-derived DHA in the food and biomedical sectors. The illustration highlights its incorporation into various food products such as bread, soft drinks, soups, dairy, and pasta, enhancing their nutritional value. Additionally, it showcases the biomedical benefits of DHA, including antioxidant, anti-inflammatory, antihypertensive, antimicrobial, heart health, eye health, and anti-cholesterol effects.

Omega-3 bioavailability is significantly affected by the food matrix; for example, nano-emulsion technology combined with yogurt as a carrier has been shown to significantly increase DHA levels when used as a carrier in a food. In the field of aquatic feed ingredients, *Schizochytrium* dry biomass is considered a promising replacement for fish oil, fishmeal, and vegetable oils, traditionally used as aquatic feed ingredients. In addition, it affects the levels of PUFAs in the tissues and improves the antioxidant capacity, the immune response, and the disease resistance of fish seedlings as well as their quality (94, 95). Studies have demonstrated that encapsulated DHA can be incorporated into milk, yogurt, and cheese products to enhance the nutritional profile of each product without altering its flavor or texture (Figure 3). DHA- and EPA-rich oil derived from *Schizochytrium* sp. can be integrated into a diverse array of food products. A few of these products include dairy and cheese, plant-based dairy replacements, spreads, salad dressings, breakfast cereals, nutritional supplements, weight management products, functional foods, baked goods, cereal bars, cooking oils, non-alcoholic drinks, infant formula, and processed grain products. The potential of microalgae to enhance the sensory qualities of food while also driving innovation and scientific progress in the fields of nutrition and food technology is emphasized by such applications.

The inclusion of *Dunaliella salina* into pasta has been demonstrated to enhance its nutritional value by enhancing its mineral, phytochemical, and unsaturated fatty acid content due to its increased nutritional value. It is also utilized in infant formulas to support neurological development and overall health. Furthermore, supplementing the diets of livestock and poultry with DHA-rich microalgae enhances the omega-3 content in meat, milk, and eggs, thereby improving their nutritional quality.

## 6 Conclusion and future prospects

DHA is an essential omega-3 fatty acid. It helps improve brain function, supports heart health, and reduces inflammation. However, adding DHA directly to foods is difficult because it breaks down easily and does not mix well with water. This can shorten the product's shelf life, create bad tastes, and reduce its health benefits. To solve these problems, scientists have developed methods to keep DHA stable and make it easier to add it to different foods. In recent years, biotechnological advances have led to the development of functional foods that contain stable encapsulated DHA. The use of encapsulation technologies has significantly enhanced the stability and bioavailability of DHA in functional foods. These delivery systems protect DHA from oxidation and improve its absorption in the gastrointestinal tract, thereby maximizing its health benefits (96). Recent advances have highlighted the use of biopolymeric carriers, such as alginate, chitosan, whey protein, and starch derivatives. These carriers are valued for their biocompatibility, sustainability, and controlled-release profiles, making them suitable for functional food applications (97, 98). Additionally, the growing trend toward plant-based and “clean label” formulations has spurred interest in natural polymers and green encapsulation techniques to meet consumer demands for minimally processed, additive-free products (99).

However, ensuring the long-term oxidative stability of DHA throughout processing, storage, and digestion in the gastrointestinal tract presents a considerable challenge. Optimizing encapsulation parameters, including particle size, surface charge, and matrix composition, is essential to prevent the premature release and degradation of DHA (100). Encapsulation systems must be carefully designed to maintain the organoleptic qualities of the final product. Moreover, the bioavailability and bioaccessibility of encapsulated DHA under simulated gastrointestinal conditions require systematic investigation as some encapsulation systems enhance bioavailability, while others may hinder DHA release at the absorption site (101). Exploring the release kinetics and interactions of encapsulated DHA within food matrices remains crucial for future research studies (96). Additionally, most studies to date have been conducted under laboratory conditions; thus, large-scale validation and cost-effectiveness analyses are essential for commercial translation of encapsulated DHA technologies (102).

Although substantial advancements have been achieved in DHA encapsulating functional food applications, future research should prioritize enhancing oxidative stability, improving bioavailability, preserving sensory attributes, scaling up industrial processes, and investigating sustainable production methods. The integration of biotechnological innovations, such as precision fermentation, genetically engineered microorganisms, and bio-inspired encapsulation systems, presents promising opportunities to enhance the production and stability of DHA. For example, microbial biosynthesis of DHA using engineered microalgae could offer a sustainable and scalable alternative to traditional fish oil sources. Integrating biotechnology with cutting-edge encapsulation methods could lead to next-generation functional foods with enhanced health advantages. DHA has been shown to be effective *in vitro*, but *in vivo* studies have been limited, making it difficult to demonstrate its efficacy. Additionally, the molecular mechanisms through which encapsulated DHA confers health benefits remain inadequately understood and necessitate further exploration. A significant challenge also lies in the high production costs and scalability of encapsulation technologies. Multidisciplinary collaborations among food scientists, biotechnologists, and industry stakeholders are essential to bridge the gap between laboratory research and practical commercial applications to increase the accessibility of these functional foods.
